# Learning Explainable Time-Morphology Patterns for Automatic Arrhythmia Classification from Short Single-Lead ECGs

**DOI:** 10.3390/s21134331

**Published:** 2021-06-24

**Authors:** Hyeonjeong Lee, Miyoung Shin

**Affiliations:** Bio-Intelligence & Data Mining Laboratory, School of Electronic and Electrical Engineering, Kyungpook National University, Daegu 41566, Korea; leehj1224k@gmail.com

**Keywords:** arrhythmia classification, atrial fibrillation (AF), electrocardiogram (ECG), convolutional neural network (CNN), deep learning

## Abstract

Automatic detection of abnormal heart rhythms, including atrial fibrillation (AF), using signals obtained from a single-lead wearable electrocardiogram (ECG) device, is useful for daily cardiac health monitoring. In this study, we propose a novel image-based deep learning framework to classify single-lead ECG recordings of short variable length into several different rhythms associated with arrhythmias. By transforming variable-length 1D ECG signals into fixed-size 2D time-morphology representations and feeding them to the beat–interval–texture convolutional neural network (BIT-CNN) model, we aimed to learn the comprehensible characteristics of beat shape and inter-beat patterns over time for arrhythmia classification. The proposed approach allows feature embedding vectors to provide interpretable time-morphology patterns focused at each step of the learning process. In addition, this method reduces the number of model parameters needed to be trained and aids visual interpretation, while maintaining similar performance to other CNN-based approaches to arrhythmia classification. For experiments, we used the PhysioNet/CinC Challenge 2017 dataset and achieved an overall F_1_NAO_ of 81.75% and F_1_NAOP_ of 76.87%, which are comparable to those of the state-of-the-art methods for variable-length ECGs.

## 1. Introduction

Cardiac arrhythmia, which is characterized by a set of abnormal heart rhythms and erratic heartbeats, is associated with several cardiovascular diseases [1]. As a common type, atrial fibrillation (AF) occurs when the atria fail to contract properly, thereby increasing the risk of stroke, hospitalization, heart failure, and death [2,3,4,5]. Thus, an accurate and effective detection of arrhythmias, including AF, is essential for the early diagnosis of cardiovascular diseases and their clinical treatments to prevent serious complications [6,7,8].

The electrocardiogram (ECG) is a common and convenient tool that is widely used to monitor cardiac abnormalities in a noninvasive manner by measuring the heart’s electrical activity. A normal heartbeat of an ECG signal generally comprises three parts, namely, the P wave representing atrial depolarization, the QRS complex standing for ventricular depolarization, and the T wave corresponding to ventricular repolarization [9]. In addition, AF is characterized in an ECG by the absence of the P wave, the presence of a small oscillation (i.e., fibrillatory f waves) in the TQ interval, and an irregular inter-beat timing (i.e., R–R interval) caused by rapid and irregular atrial contraction. Thus, the regularity of the morphology and the rhythm of heartbeats in ECG signal is primarily analyzed for arrhythmia detection.

Most existing AF detection methods based on ECG fall into one of the two following categories: (1) atrial activity-based methods [10,11,12], which focus on identifying the absence of P waves and the presence of f waves; and (2) ventricular response-based methods [13,14,15], which focus on examining the irregularities of the R–R interval. The former tends to show better performance than the latter but is vulnerable to noise. On the contrary, the latter is relatively robust to noise, but requires long-length ECG recordings and is often confused with other non-AF arrhythmias that exhibit irregular R–R intervals. Therefore, it appears inevitable to consider the combination of both atrial activity- and ventricular response-based analyses for more sophisticated AF detection [16,17,18].

To date, various types of morphological or statistical features manually extracted from ECG signals in the time domain, frequency domain, or nonlinear and transformed domain have generally been used in conventional machine learning classifiers for arrhythmia detection and classification [19,20,21,22,23,24,25,26]. Although some of these have shown acceptable performance in several studies, hand-crafted features are difficult to generalize in other situations.

At present, end-to-end deep learning techniques, such as convolutional neural networks (CNNs) and recurrent neural networks (RNNs), are being widely applied to meet the need for automated feature selection and classification of ECG signals [27,28,29]. These techniques are particularly attractive because of their ability of automatically learning inherent well-distinguishable features from raw inputs without relying on domain knowledge or expert intervention. Thus, the long short-term memory (LSTM) or the convolutional recurrent neural network (CRNN) is frequently used in recent ECG classification tasks [14,30,31,32]. These models facilitate the extraction of global time-dependent features (i.e., time-varying dynamics between multiple beats) with self-recurrent connections but have the disadvantage of taking a long training time and incurring conversion overhead. On the contrary, CNN can learn local characteristics (i.e., morphological traits of each beat and its nearby beats) effectively in a relatively short time [33,34,35,36]. However, despite their fully automatic learning abilities and impressive performance, CNNs tend to perform well as the network deepens; hence, they often lead to a tremendous increase in the number of training parameters [37]. Consequently, understanding learned features via internal complex architectures of deep learning models intuitively has become a difficult issue.

In addition, due to the advance and spread of wearable devices, there is significant demand for the utilization of single-lead ECG signals of variable length in various contexts. In spite of the considerable achievements in earlier single-lead ECG studies [38,39,40], a reliable AF detection in real-world scenarios remains a challenge for the following reasons. First, single-lead ECG signals tend to be unstable. That is, they are very sensitive to the environment and contain limited information compared to the conventional 12-lead ECG signals. Second, the AF detection based on short-length ECG recording (e.g., 30 s to 1 min) is preferable for early arrhythmia diagnosis. Third, AF is an episodic event and has characteristics similar to some other arrhythmias [26]. Finally, deep neural networks are often not well trained for AF detection because of the highly class-imbalanced dataset (i.e., the dataset often has much smaller AF samples than normal samples).

To address the abovementioned issues, herein we propose a novel and efficient deep learning framework that employs short single-lead ECG recordings of variable length for arrhythmia classification. By transforming a variable-length one-dimensional (1D) ECG signal into a fixed-size two-dimensional (2D) time-morphology representation and feeding it into the beat–interval–texture CNN (BIT-CNN) model, we aim to investigate the understandable characteristics of beat shape and inter-beat patterns over time for automatic arrhythmia classification. This approach is different from that of other state-of-the-art deep learning-based ECG classification studies in which a 1D signal is directly applied or converted to a 2D spectrogram (i.e., spectro-temporal representation).

Using our approach, we attempted to (1) identify distinguishable features that reflect the shape and rhythm of heartbeats appearing in an arbitrary short time; (2) develop the BIT-CNN model capable of achieving a good classification performance with a relatively small number of training parameters; and (3) facilitate an intuitive understanding of class-specific and layer-specific activations of multiple features (filters) by the BIT-CNN model. 

The main contributions of this study can be summarized as follows:

- We use a fixed-size 2D representation, called consistent-sized ECM (CS-ECM) built from a short single-lead 1D ECG signal of variable length. The CS-ECM was designed to handle the ECGs of a variable short-length (30 s to 1 min) by modifying the original ECM [41] via size adaptation. This allows the shape and the rhythm of heartbeats over time to be jointly expressed as a 2D image of consistent size and fed into the input for BIT-CNN-based learning.
- We propose a novel BIT-CNN architecture characterized as (1) consisting of three types of convolution filters with different shapes to learn the significant characteristics shown within each beat and between multiple beats; (2) utilizing 1 × 1 convolution filters to effectively summarize the channel dimension of feature maps and reduce the number of parameters to be learned; (3) applying dual (max/average) pooling operations to reduce the spatial channel dimension of feature maps; and (4) employing a spatial and channel attention mechanism to enhance the expressive power of representation vectors by selectively focusing on the salient parts.
- With the proposed methodology, we develop an arrhythmia classification model to classify short single-lead ECG recordings into four different rhythms, namely normal, AF, other arrhythmias, and too noisy. In addition, for an in-depth understanding of the trained model, we visually inspect the class-specific and layer-specific filter activations of the input ECG signals.

The remainder of this paper is organized as follows. In Section 2, we describe the details of the proposed methodology that employs the CS-ECM images as the input to the BIT-CNN model for feature learning and classification of variable-length ECG signals. Section 3 presents the experimental results and performance comparison. Finally, conclusions and some discussions are given in Section 4.

## 2. Methodology

### 2.1. Overview

**Figure 1:**
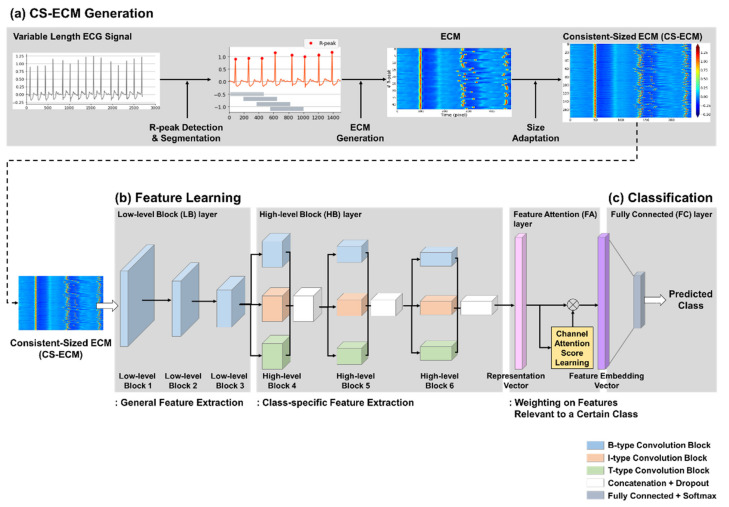
Overall workflow of the proposed learning framework for classifying the short ECGs of variable length. (**a**) CS-ECM generation; (**b**) feature learning; (**c**) classification.

The proposed learning framework based on the BIT-CNN model, shown in Figure 1, consists of three different stages: CS-ECM generation, feature learning, and classification stages. In the CS-ECM generation stage, each 1D ECG recording of arbitrary length is transformed into a fixed-size 2D ECM matrix. Because CNN requires input data to be of the same size, we constructed CS-ECMs that are consistent in size for variable-length ECG recordings without adding redundant values such as zeros or a truncation step that can cause information loss. Next, the feature learning stage aims to learn distinguishable feature embedding vectors from a given collection of CS-ECMs based on the BIT-CNN architecture. Finally, the classification stage is used to predict the class probabilities from the learned embedding vectors. This model is trained with the back-propagation algorithm until the stopping condition is satisfied. Each of the three stages of the proposed methodology is explained in detail in the following subsections.

### 2.2. Consistent-Sized ECM for Handling Variable-Length ECGs

The electrocardiomatrix (ECM) is a 2D representation of ECG signals, first introduced in [41]. It represents the shape and the rhythm of heartbeats in the form of a 2D matrix by splitting a given 1D ECG signal into a series of short segments of equal length containing two adjacent QRS complexes and aligning them vertically based on the first R-peak positions of the segments. In earlier studies, the ECMs were mainly used to monitor long-term ECG signals and/or detect their abnormalities by visual inspection [42,43].

**Figure 2:**
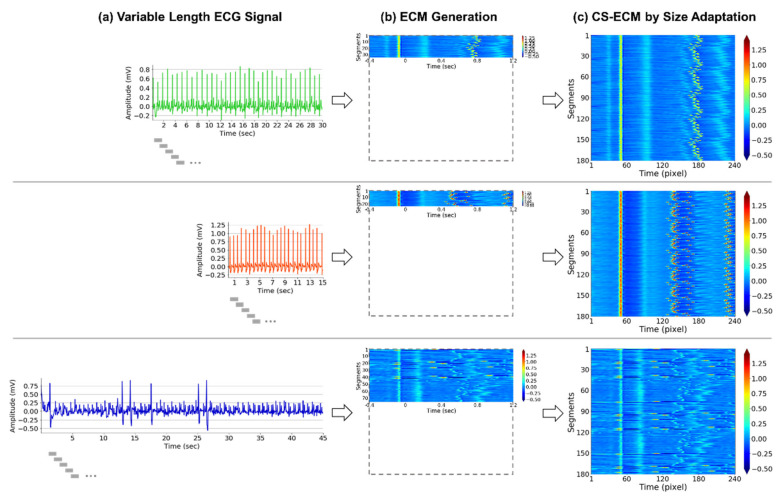
CS-ECM generation of short variable-length ECG signals. (**a**) Raw ECG signal and corresponding short segments (gray bars); (**b**) original ECM generated by vertically aligning short segments based on the first R-peaks; and (**c**) CS-ECM obtained by applying size adaptation on the original ECM.

In the present study, we utilized the modified ECMs to handle short variable-length ECG signals for arrhythmia classification based on the BIT-CNN model. Specifically, we adjusted the size of conventional ECMs produced from the ECG signals of different lengths to make it consistent because CNN requires all inputs to be of the same size. Thus, the consistent-sized ECMs (CS-ECMs) were generated by applying an additional step of size adaptation for the original ECMs (refer to Figure 2). Here, the original ECMs were produced in the same manner as in [41], except for changing the parameter values. For the size adaptation, if a given ECG signal is of a short length (i.e., under 1 min) such that its segments are too few to fill the ECM of a specific size, we repeatedly pad them in series until the ECM is filled, eventually producing the CS-ECM. Conversely, if a given ECG signal is of a long length (i.e., longer than 1 min) such that its segments are too many to fill the ECM of a specific size, we perform segmentation to divide the ECG signal into several short signals of 1 min length or less and generate multiple CS-ECMs from those short signals.

For this purpose, each ECG recording was split into segments of 1.6 s length, where the *i*-th segment starts 0.4 s before and ends 1.2 s after the fiducial point of the *i*-th detected R-peak. The average R–R interval (time between two adjacent R-peaks) in our experimental dataset was 0.8 s; thus, each segment can contain sufficient information about the beat morphology and the rhythm (i.e., R–R interval) between the corresponding and subsequent beats. The length of each segment (i.e., width of each CS-ECM) was reduced from 480 (300 Hz) to 240 (150 Hz). The height of each CS-ECM was fixed at 180, which is the maximum number of segments in an ECG recording in our dataset. Finally, we obtained the fixed-size CS-ECM with dimensions of 180 × 240 pixels.

**Figure 3:**
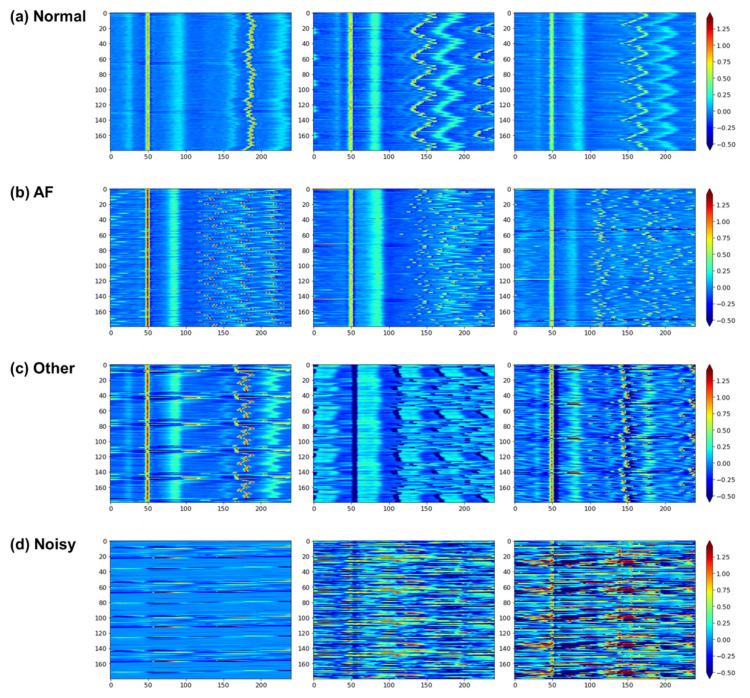
Examples of CS-ECMs with a fixed size (180, 240). Panels (**a**–**d**) correspond to the classes of normal, AF, other abnormal rhythms, and too noisy, respectively. The magnitude (voltage) of ECG signal is indicated by the color bar, denoting low and high values with the blue and red colors, respectively.

Figure 3 illustrates some CS-ECM characteristics for each of the four classes. Figure 3a illustrates the CS-ECM of the normal sinus rhythm (normal class). The beat morphologies around the fiducial R-peaks were similar to each other, with some regularities. The narrow P waves and the wide T waves were clearly visible before and after the R-peaks. Moreover, the R–R interval smoothly changed in the form of waves without breaking. Figure 3b shows the CS-ECM of atrial fibrillation (AF class). Here, it is difficult to find P waves. The R–R intervals changed irregularly while showing the salt-and-pepper noise pattern. They also looked shorter than in other classes, indicating that the heart beats faster. Figure 3c depicts the CS-ECM of other abnormal rhythms except AF (other class). Here we can see various types of patterns that are not shown in the previous two classes (normal, AF). One of these was generated by a ventricular ectopic beat. A few irregular patterns appeared between consecutive regular patterns. Because this class contains multiple types of arrhythmia, additional various patterns can appear, including unique P-QRS-T complex patterns, irregular R–R intervals but different from that of AF class, T wave loss, etc. Figure 3d shows the CS-ECM of a very noisy rhythm (noisy class). Here, it is difficult to visually determine the P-QRS-T complexes, or there are many noise parts within one recording, or the distinction between the noise part and the other class part is ambiguous.

### 2.3. The Proposed BIT-CNN Model

#### 2.3.1. Model Architecture

**Figure 4:**
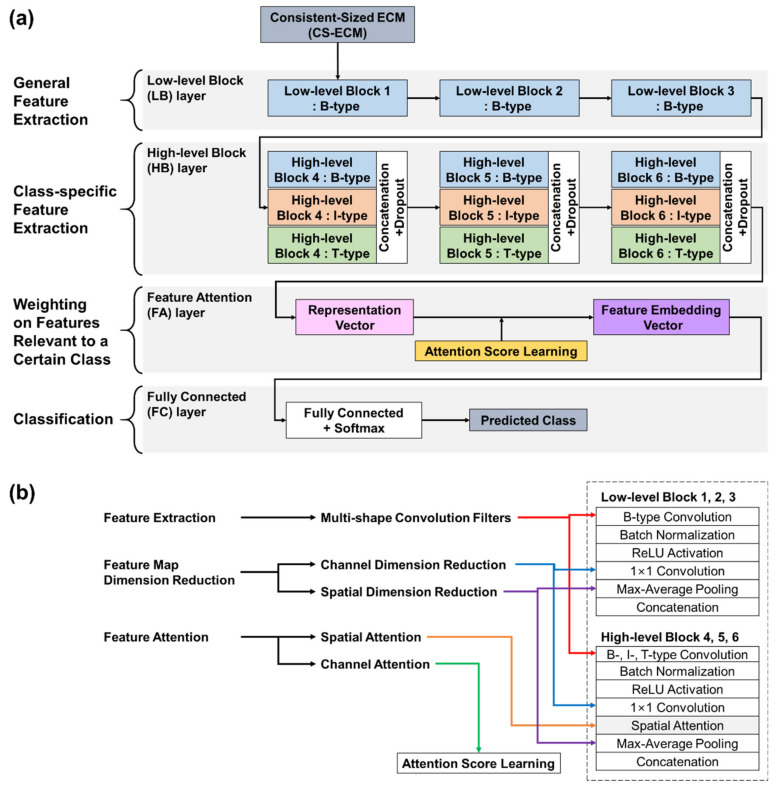
The proposed BIT-CNN model: (**a**) architecture and (**b**) primary functional modules.

The proposed BIT-CNN model consists of four different layers, namely, low-level block (LB) layer, high-level block (HB) layer, feature attention (FA) layer, and fully connected (FC) layer (refer to Figure 4a).

The LB layer is composed of three low-level blocks referred to as blocks 1 to 3 and designed to well extract the general (class-independent) features from the CS-ECM images, such as R-peaks, P waves, and T waves. Specifically, each block contains B-type convolution, batch normalization, ReLU activation, 1 × 1 convolution, max-average pooling, and concatenation. The output of each block in the LB layer is given as a single feature map obtained through the channel-wise concatenation of max pooling and average pooling.

The HB layer consists of three super-blocks referred to as blocks 4 to 6. Each super-block includes three high-level blocks, followed by a dropout. This layer is designed to extract more complex class-specific features, including ectopic beats and irregular R–R interval patterns. Each high-level block contains one of three different types (i.e., B-, I-, and T-types) of convolution, batch normalization, ReLU activation, 1 × 1 convolution, spatial attention, max-average pooling, and concatenation. The configuration of each high-level block is similar to that of low-level blocks, except for the spatial attention between 1 × 1 convolution and two poolings. After passing through three high-level blocks in parallel, the dropout is applied to prevent overfitting.

In the FA layer, the representation vectors obtained from the HB layer are associated with the channel attention scores separately learned from the multi-layer perceptron (MLP) to produce the eventual feature embedding vectors of the input CS-ECMs. To do this, the HB layer output is given to the two operations of global average pooling (GAP) and global max pooling (GMP) in parallel, and the two outputs are concatenated to have the representation vectors for the CS-ECM inputs. Then, by associating them with the attention scores learned via MLP, we finally obtain the FA vectors of the CS-ECMs.

The FC layer aims to predict the probabilities of the target classes from the feature embedding vectors learned through the abovementioned layers using the softmax function.

The primary function modules of the BIT-CNN model are illustrated in Figure 4b, and their characteristics are depicted in the following subsections.

##### Multi-Shape Convolution Filters

In this study, we utilized three types of convolutional filters with different shapes, which are referred to as B-, I-, and T-type filters, to extract a variety of more characteristic patterns from CS-ECMs. Considering the CS-ECM regions where each filter covers and distinctive patterns of extracted features, we grouped and named them as follows.

The B-type convolution filter indicating a filter for beat has a kernel of size 1 × K to consider the K timesteps of each CS-ECM segment at a time. This filter focuses on finding the beat morphological features occurring in each ECM segment, such as the existence of P waves, position of R-peaks, etc. This operation is similar to that of the general 1D filter that conducts a 1D convolution on a raw ECG signal. Note that in the LB layer of the proposed BIT-CNN model, only the B-type convolution filters were used to capture the valid features of the beat morphology in each CS-ECM segment. Repeating the B-type convolution with pooling several times leads to a situation in which the R-peak positions are almost aligned in the reduced feature map, even if they are initially misaligned in the raw CS-ECM image.

The I-type convolution filter representing a filter for the interval of beats has a kernel of size K × 1, which considers a single timestep of K segments in the CS-ECM at a time. This filter focuses on finding the beat interval-related features that consistently appear in a certain time range of the ECG signal exhibited in the CS-ECM. For example, it can be used to examine whether the P wave continuously appears, whether the T wave varies over time, etc.

The T-type convolution filter, which stands for texture-type filter, has a square kernel of size K × K, to extract an extensive range of patterns by considering both the morphological and temporal variations shown in similar timesteps of multiple adjacent ECM segments. This filter is effective in finding features occurring in a wide area, such as variations in the length of R–R intervals that often appear irregular in the AF class, but in wave form in the normal class.

In our implementation, the kernel size K was set to 5 in all convolution filters. The numbers of filters used in blocks 1 to 6 are 32, 64, 128, 64 × 3, 128 × 3, and 256 × 3, respectively, and 1568 filters in total. Each channel of a feature map is the output of the convolution with a filter; hence, the number of channels in the feature map is equal to the number of convolutional filters in each block. For example, 32 filters in block 1 convolve with an input matrix, producing a feature map of 32 channels.

##### 1 × 1 Convolution for Channel (Depth) Dimension Reduction

A filter in the form of 1 × 1 × (the number of extracted features) was first introduced in [44] as a strategy for reducing the number of channels of a feature map (i.e., reducing depth dimension). This is understood as a process of combining features found by multiple convolution filters at all locations in the feature map by weighting them with channel importance. In this respect, the 1 × 1 convolution is an efficient method of compressing the feature map channel (depth), resulting in the reduction of the number of parameters to be learned.

Referring to the clinical characteristics of the ECG signals previously known for cardiac diseases, the three following regions of the CS-ECMs need to be examined: (1) the area before the first R-peak containing information on the P wave, etc.; (2) the area after the first R-peak, including information on the T wave, interval between two adjacent R-peaks, etc.; and (3) the area around the second R-peak containing a variation of the R–R interval length, etc. Thus, under the assumption that the activation of some filters (e.g., P-wave-related features) is important in the first area, but may not be meaningful in other areas, and vice versa, we set the number of filters for each block as a multiple of 3 to combine features by selectively considering the three regions with different importance. The numbers of 1 × 1 convolution filters in blocks 1 to 6 were chosen as 3, 6, 12, 18, 24, and 30, respectively. Thus, a feature map of 32 channels in block 1 is aggregated into a feature map of 3 channels using three 1 × 1 convolution filters. Similarly, the other blocks work in the same manner as block 1.

##### Dual Pooling for Spatial Dimension Reduction

The pooling operation is a means of reducing the dimension of the width and the height of a feature map by downsampling components contained in the feature map. Among several types of pooling, max-pooling focuses on capturing the most discriminative values in the local regions of a feature map, which usually corresponds to prominent features that are vulnerable to noise. By comparison, average-pooling focuses on finding the representative values of local regions in a feature map by taking the average of all the values in each local region while preserving the universal information of the feature map. Thus, in the proposed BIT-CNN model, we used max-pooling and average-pooling together to take advantage of both cases by retaining the highest and average information.

**Figure 5:**
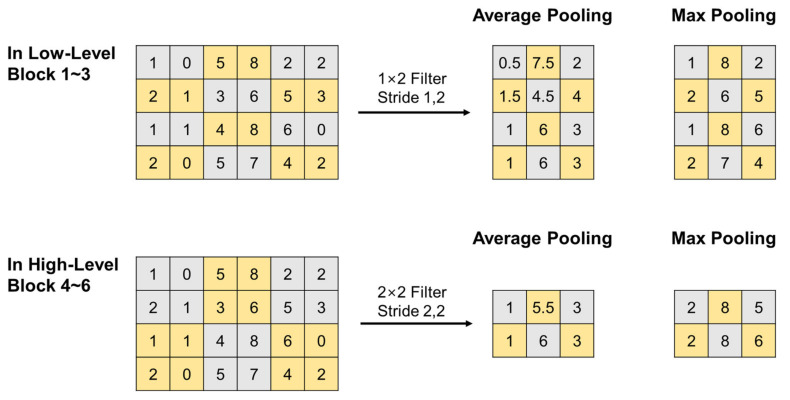
Pooling operations different in low- (**top**) and high-level blocks (**bottom**).

**Figure 6:**
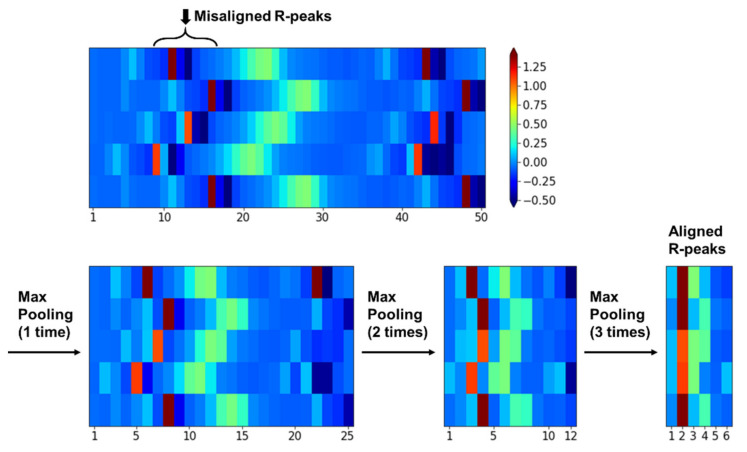
Illustrating the effect of a series of the max pooling operations in the low-level blocks.

The pooling operations were applied differently in low- and high-level blocks (refer to Figure 5) because they employ different combinations of convolution filters. In the high-level blocks, the pooling operation with a stride of 2 was applied in the same manner as that commonly used in a 2D CNN. That is, both the width and the height of a feature map are reduced by half. By comparison, in low-level blocks that only use B-type filters, the pooling operation led to a decrease only in the width of CS-ECM because the convolution operation considers one segment of CS-ECM (i.e., each row) at a time. The pooling operation repeatedly conducted for several times in the LB layer made it robust against the positional variation of the features extracted in the nearby R-peak region, even if the initial R-peaks were not correctly aligned in the raw CS-ECM images, as illustrated in Figure 6.

##### Spatial Attention in the HB Layer

Research for the attention mechanism in deep learning has been mainly conducted in RNN models such as LSTM, and attention methods applicable to CNN have recently been actively studied [45,46]. Because patterns related to multiple classes may be mixed within an ECG recording, it is necessary to pay more attention to patterns related to a particular class. In the current study, the spatial attention method used in [47] is applied after the 1 × 1 convolution. Spatial attention scores can be computed through gradient-based method by applying average- and max-pooling operations along the channel axis of the previous feature map and performing a convolution. We apply the spatial attention mechanism to high-level blocks (blocks 4–6) to concentrate on the salient features to separate the classes.

##### Channel Attention Score Learning

In the FA layer, we first performed global average pooling (GAP) or global max pooling (GMP) to extract a single value from each channel of the feature map produced in the last HB block. By repeating this over the channel axis of the feature map, we obtained two feature vectors from GAP and GMP, whose length was equal to the number of channels in the feature map. Then, we concatenated the two feature vectors produced from GAP and GMP to obtain a single representation vector.

In addition, we attempted to learn an attention score of the representation vector. Each component of the attention score vector corresponds to the significance of each channel of the feature map produced in the last HB block. To learn the attention scores of the channels, we used the multi-layer perceptron (MLP) with one hidden layer. The representation vector weighted by the channel attention scores eventually becomes our feature embedding vector, which was then fed to the FC layer to perform a final classification. As the feature map dimension from block 6 was 180, both outputs of GAP and GMP were 180, and the length of their concatenated representation vector and the final feature embedding vector was 360.

#### 2.3.2. Model Training

**Figure 7:**
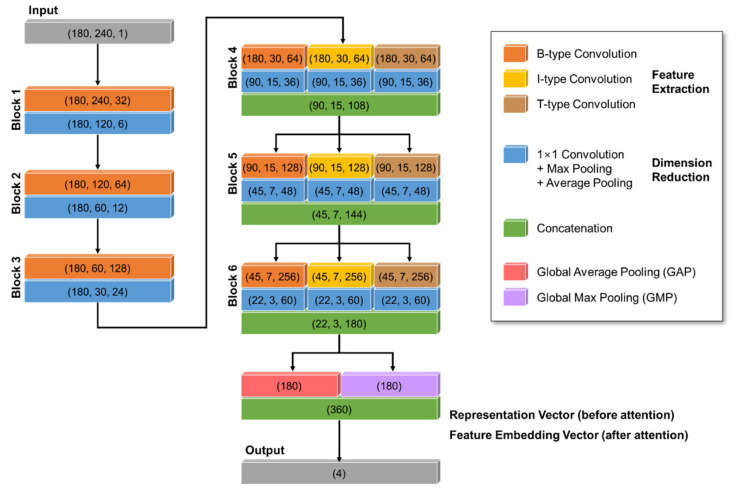
Detailed description of the proposed BIT-CNN configuration. The output shape of each operation is expressed in the form of (height, width, channel) or (length of vector). For simplicity, some layers without size changes are not shown here.

To train the proposed BIT-CNN model, we used the adaptive moment estimation (Adam) optimizer, which is a variant of the stochastic gradient descent algorithm, with a learning rate of 0.001. All training data were divided into mini-batches. The batch size was set to 16 to speed up the training process. We also used categorical cross-entropy as the loss function under the standard setting given by Keras with a Tensorflow backend. We trained the BIT-CNN for a maximum of 100 epochs, but it was stopped after 20 epochs if there was no improvement. The detailed structure and size of the receptive fields for each layer in our implementation are given in Figure 7. In this figure, some modules (e.g., batch normalization, attention modules, and dropout) that do not affect the size change of the receptive fields were excluded for better understanding. Finally, our BIT-CNN model takes a total of 2,015,043 parameters into account including 2,011,907 trainable parameters.

## 3. Experimental Results and Discussion

In this section, we investigate the effectiveness of BIT-CNN in terms of the classification performance and interpretability. We validate our approach using a publicly available dataset and compare the classification performance with those of the other state-of-the-art methods. We also discuss the effectiveness of components in our network. Finally, we examine the internal operations and hidden informative relations between the CS-ECM images (input) and the classification results (output) using some visualization techniques. All experiments were implemented in Python 3.6.4 using Keras with a Tensorflow backend and run on an NVIDIA GeForce RTX 2070 GPU and 16 GB RAM.

### 3.1. Dataset

The publicly available dataset provided by PhysioNet/Computing in Cardiology (CinC) Challenge 2017 [48] was used in this study. This dataset was originally acquired by individuals with AliveCor portable lightweight devices that allow personal heart monitoring at all times and in all locations. The dataset includes 8528 single-lead fingertip ECG recordings of a short duration lasting between 9 and 61 s (30 s on average) with a sampling frequency of 300 Hz. All recordings were labeled by experts with one of the four categories, resulting in the following count: 5076 normal (normal sinus rhythm); 758 AF (atrial fibrillation); 279 noisy (too noisy to be recognized); and 2415 other (abnormal rhythms that do not belong to AF or noisy) recordings. The statistical summary of the dataset is given in Table 1.

### 3.2. Evaluation Method

For evaluation, we used a stratified five-fold cross-validation in which each fold preserves the original proportion of each class. For each fold, 20% of the data was considered the test set, and the remaining 80% was divided into 90% for training and 10% for validation. Thus, from original dataset given in Table 1, we have approximately 1015 normal, 151 AF, 483 other, and 56 noisy recordings in each fold, which shows a severe class imbalance problem.

To lessen the impact of this problem, in each fold, oversampling was applied to the minor classes (i.e., AF, other, and noisy) to obtain 2500, 3000, and 1000 recordings, respectively, and undersampling was applied to the major class (i.e., normal) to obtain 4000 recordings. These values were empirically chosen based on the assumption that each class may need a different number of distinguishable features to be learned for a better classification. For example, the normal and AF classes were relatively easy to distinguish, whereas differentiating the normal and other classes was more difficult. Moreover, many sub-classes belonged to the other class; thus, more features needed to be learned for other detection.

The classification performance was assessed using the F1 score (i.e., a harmonic mean of precision and recall) for each class, which is given as follows:

F_1_ (%) = (2 × Precision × Recall)/(Precision + Recall) × 100 (1)

Precision = TP/(TP + FP), Recall = TP/(TP + FN) (2)

where TP, FP, and FN are the true positives, false positives, and false negatives, respectively. Here the precision (also called the positive predictive value, PPV) and recall (i.e., sensitivity) are derived from the confusion matrix obtained by aggregating the classification results of five folds. The two metrics are often in a trade-off, which means that increasing the precision typically decreases the recall, and vice versa.

We also used the indicators guided by PhysioNet/CinC Challenge 2017 [48], given as:

F_1_NAO_ = (F_1Normal_ + F_1AF_ + F_1Other_)/3 (3)

F_1_NAOP_ = (F_1Normal_ + F_1AF_ + F_1Other_ + F_1Noisy_)/4 (4)

These metrics define the overall evaluation score as the average of the individual F1 scores for the normal, AF, and other classes, excluding or including the noisy class.

### 3.3. Performance Evaluation and Comparison

Using the proposed BIT-CNN model for arrhythmia classification, we achieved an average F_1_NAO_ of 81.75% and F_1_NAOP_ of 76.87% with five-fold cross-validation. Specifically, it was approximately 90% for the normal class, 81% for the AF class, and 74% for the other class. Table 2 presents more detailed results for the four classes.

As expected, the BIT-CNN model worked best in predicting the normal class. It was also good at distinguishing AF rhythms from normal rhythms because only a small portion of normal (23 out of 5076 samples = 0.45%) and AF (29 out of 758 samples = 3.83%) samples were incorrectly classified to each other. On the contrary, it appeared to have difficulty in differentiating between other and normal classes, showing a misclassification of 6.97% (i.e., 354 out of 5076) normal samples to the other class and 21.7% (i.e., 524 out of 2415) other samples to the normal class. Similarly, approximately 13% of AF samples was misclassified as the other class. In this dataset, the other rhythm indicates a group of diverse abnormal non-AF rhythms, potentially including many subgroups with small numbers of samples. Thus, it is conjectured that the poor performance for the other class may be caused by the difficulty of learning sufficient representative features to differentiate other from the remainder.

It is worth noting that most of previous studies on the four-class classification task using this CinC 2017 dataset have achieved the highest performance (F_1_NAO_) in the early and mid—80% [18,49,50,51]. The overall low performance probably resulted from the mixed multiple rhythms within the same recording, such as the episodic events lasting only a few seconds. Even if different rhythms or noisy parts could be found within one recording, only one class label is assigned to an entire recording in this dataset, making the features corresponding to a particular class unclear and perturbed. In addition to the high variability of the ECGs from a large number of different patients, the extreme imbalance in the four classes also makes it difficult to extract robust features for differentiating AF from noise, normal, and other types of abnormal rhythms.

For a comparison, Table 3 summarizes the performance of several published studies on the CNN-based ECG classification on the same dataset. In this table, some of the values are denoted as not available (N/A) because [52] measured the performance for each class only in terms of accuracy, and the class-specific F1 scores are not given in [53]. Overall, the proposed BIT-CNN worked better than or was comparable to the other state-of-the-art methods in terms of the average F1 scores and F1 scores for normal and AF classes. In particular, when considering only the methods that allow the variability of input signal length, our method performs well without a significant degradation of classification performance while allowing significantly easier visual interpretation compared to other studies. The performances of [52,54] are slightly better than that of the BIT-CNN; however, their classifiers cannot handle inputs of variable lengths. They require a sequence truncation step or the addition of redundant values to equalize the classifier’s input length. In contrast, our approach is applicable for signals of varying lengths via the utilization of CS-ECMs consistent in size for variable-length inputs. Ref. [55] achieved the highest performance in Table 3 by developing 16 residual convolutional blocks followed by three recurrent layers. Moreover, the recurrent layer makes their model applicable to variable-length input signals. However, it remains a challenge to interpret learned features and decision-making processes in models with many deep layers and complex structures.

Next, we compared the effectiveness of BIT-CNN in terms of model complexity. Compared to other methods having deep complex architectures with residual connections, recurrent layers, and an enormous number of parameters [53,54,55,56,57,58], the proposed BIT-CNN achieved a competitive performance without any shortcuts and by utilizing only 2,015,043 (approximately 2 million) parameters. For instance, the number of network training parameters in [52] using a simple 1D CNN was approximately 3 million, which is about a million more compared to ours. An efficient BIT-CNN structure for learning features from a comprehensive representation of ECG signal requires a small memory space.

We can also see from the table that few previous studies have explored the interpretability of the trained models until recently. In [53], the visual interpretation of the important area on a 1D waveform that supports the predicted outcome mostly is provided to aid the intuitive understanding of the model prediction. Although such a visualization approach is interesting, interpreting important areas on a 1D ECG sequence nonetheless requires expert knowledge. In addition, it is time consuming to visually inspect all of the important parts shown in the whole 1D ECG sequence. To examine the difference of spectro-temporal representations, [58] investigated the visualization of the feature map and activation in the first convolutional layer. As for the proposed BIT-CNN, several visual interpretations can be performed to capture the inner working of our model in detail, as described in the subsequent section.

To summarize, the BIT-CNN with CS-ECM input is a promising approach, particularly in terms of the two following aspects. First, the classification performance is likely to be improved when more learnable filters are added because the network effectively deals with the dimension of feature maps without being burdened by large parameter computations. Second, the compact representation of the CS-ECM is good for providing an at-a-glance overview of the variation of multiple heartbeats over time in variable-length ECG recordings. In addition, it is not only able to automatically learn features via the deep learning approach but also provides an intuitive comprehension of the important features or patterns and their location, which significantly contributes to the classification.

### 3.4. Interpretability of the Learned Features

Understanding the internal processes of machine learning models for decision making is highly important for their real application. In particular, in the AF classification problem, this understanding is critical for clinicians’ trust and clinical practice [59,60]. Unfortunately, however, deep neural networks often showing a good performance in this problem are complex black-box models, making it difficult to verify whether the decision-making process is appropriate in the clinical sense. Thus, it is desirable to develop an interpretable or explainable model that does not exhibit a significant degradation of classification performance. To address the issue of model interpretability, we examined the internal details of the trained BIT-CNN model through visualization techniques, focusing on the salient features learned through some intermediate steps of training.

#### 3.4.1. Characteristics of the Learned Feature Space in Different Layers

**Figure 8:**
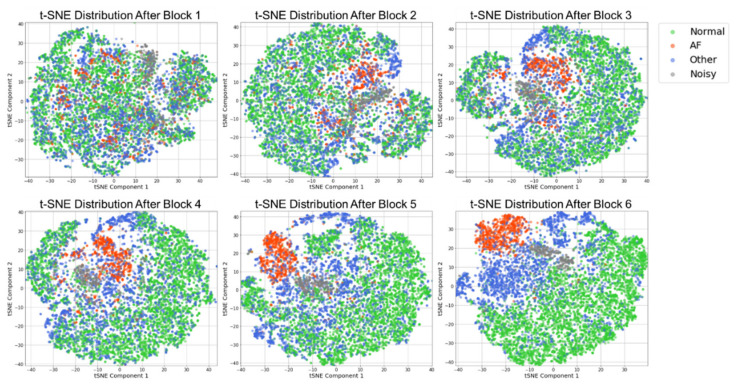
*t*-SNE visualization regarding the class-distinguishability of the learned feature vectors in blocks (1–6). For convenience, the samples belonging to each class are indicated as points with colors.

To understand the characteristics of the learned features in different layers of the BIT-CNN for AF classification, we first visualized the class-distinguishability of the latent feature vectors learned through each block of the LB and HB layers, which are shown in Figure 8. To manipulate these high-dimensional vectors for visualization, we used *t*-distributed stochastic neighbor embedding (*t*-SNE) [61] with principal component analysis (PCA) [62] and produced 2D vectors. The *t*-SNE approach is advantageous in preserving the consistency of the neighborhood distributions between high- and low-dimensional spaces. In Figure 8, for visualization, the dimension of the latent feature vectors extracted in blocks 1 to 6 was reduced using PCA with 50 principal components. *t*-SNE was then applied to obtain 2D vectors. In this figure, the distinction between the classes became more apparent as the learning deepened from blocks 1 to 6. For example, in block 1, most of the class samples have not yet been clustered, except for noisy samples that form a single cluster. As the learning progresses further in blocks 2 and 3, AF samples started to cluster while normal and other classes were still mixed together. Going further in blocks 5 and 6, the AF class was clearly observed as a cluster, while normal and other classes were shown to be better separated.

In addition, some interesting relationships among classes are inferred from Figure 8. For instance, in the high-level blocks (i.e., blocks 4 to 6), the two clusters of AF and noisy samples were close to each other, indicating that they have somewhat similar characteristics. This was presumed to be because the aforementioned AF characteristics showing an irregular R–R interval pattern due to an unpredictable heart beat may also appear in noisy samples. On the contrary, the two clusters of AF and normal classes were far from each other, implying that the distinction between AF and normal classes was relatively uncomplicated.

**Figure 9:**
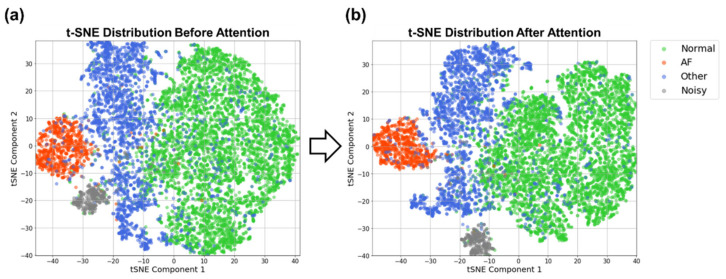
*t*-SNE visualization regarding the class-distinguishability of the learned representation vectors through block (1–6) (**a**) without channel attention and (**b**) with channel attention.

Figure 9 demonstrates the effect of channel attention on the representation vectors learned by going through blocks 1–6. In this figure, the channel attention was applied for the 360-dimensional representation vectors obtained from block 6, the result of which was visualized in the 2D space produced by *t*-SNE. After channel attention, the samples within each class were found to be more condensed, and the class boundaries became more obvious. Unfortunately, however, some normal and other samples were still mixed together. This could be due to a mixture of multiple classes within a single recording, which are thus mislabeled, or the inability of our feature embedding vectors to sufficiently differentiate the two classes.

#### 3.4.2. Layer-Specific Attention Regions for Class Distinction

To investigate layer-specific attention regions for class distinction, we applied a gradient-weighted class activation map (Grad-CAM) [63]. Grad-CAM is a generalization method of the class activation map (CAM) [64] that aims to localize the implicit attention corresponding to a particular class, which is applicable to CNN-based models by utilizing gradients. Grad-CAM creates a heatmap that highlights the important parts of the input image that are considered important (i.e., attended) in each block for class distinction. 

**Figure 10:**
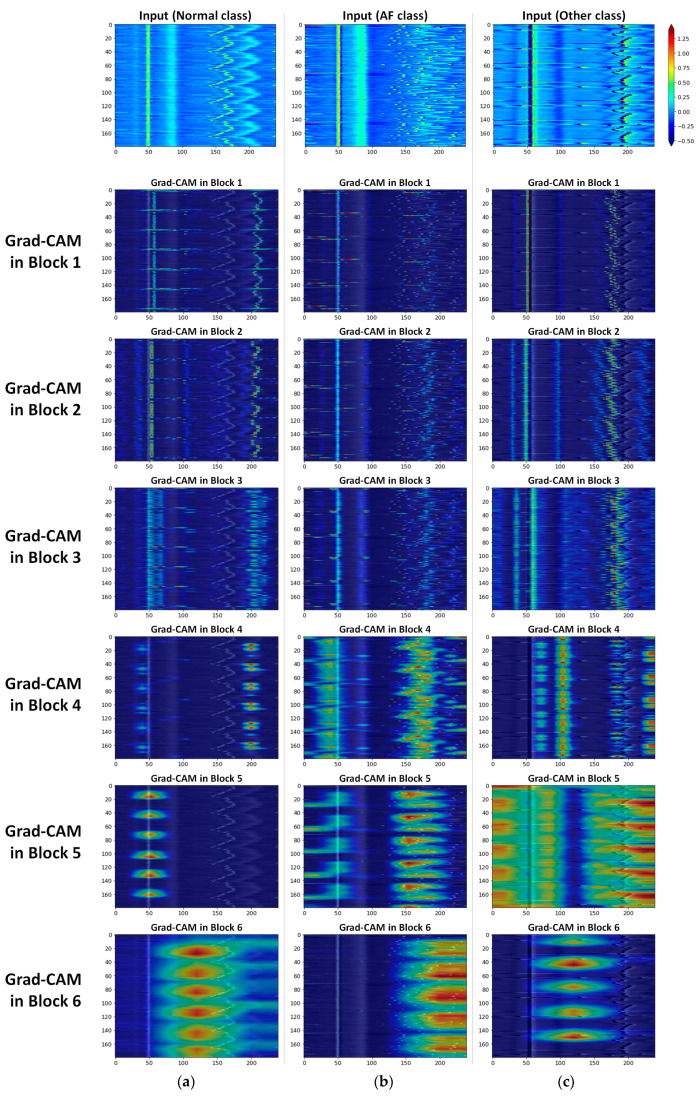
The difference in the attended regions of blocks 1 to 6 among (**a**) normal, (**b**) AF, and (**c**) other classes.

Figure 10 presents the Grad-CAM images for normal/AF/other classes to examine where the BIT-CNN model focused on each block (blocks 1–6). The colors overlaid on the input image indicate the spatial importance learned by the BIT-CNN when the ECG recording of the CS-ECM image was a normal/AF/other rhythm. In the low-level blocks (blocks 1–3) where only B-type filters were used for feature extraction, large attentions were concentrated on areas where values change rapidly, such as R-peaks and edges. In the high-level blocks (blocks 4–6), the feature maps obtained by three filter types were combined to obtain one Grad-CAM heatmap. Unlike focusing on small and local features in low-level blocks, the BIT-CNN recognizes and concentrates on a large, global patterns or feature sets that appear across a wide area in the high-level blocks.

Interestingly, in Figure 10a,b, the salient areas in the CS-ECM image of the AF class were significantly different from those of the normal class. In particular, the Grad-CAM in block 4 showed highlights concentrated in the vertical areas, where no P waves existed. Another focusing area was found on the right side of the heatmaps in blocks 4 to 6, which appeared as scattered sand-like patterns caused by the irregularly occurring R-peaks. Through the visualization, it is demonstrated that our BIT-CNN considered two important characteristics of AF in ECG signals, that is, the absence of the P wave and the irregular R–R intervals.

In contrast, in the CS-ECM image of the other class, shown in Figure 10c, the regions corresponding to the P wave, QRS complex, and T wave showed reverse values, unlike the examples in the normal and AF classes. This result can be attributed to the inverted ECG signal. The highlighted regions shown in heatmaps for the low-level blocks (i.e., blocks 1–3) indicated that the BIT-CNN model considered the aforementioned situation when capturing features for classification without preprocessing the inverted signals. The attentions in the high-level blocks (i.e., blocks 4–6) for the other class also differed from normal and AF classes. Although we do not know which type of arrhythmia is in the other class, it is understandable that the three types of filters looked at a wide range of areas before and after R-peaks, particularly in Block 5, when classifying other class.

#### 3.4.3. Filter-Specific Attention Regions for Class Distinction

Here we investigate the difference in filter-specific attention regions for class distinction. Because three types of convolutional filters (i.e., B/I/T-type filters) were employed in the high-level blocks (i.e., blocks 4–6), we disentangled the Grad-CAM heatmap in Section 3.4.2 by filter type to visually examine the important areas where each filter type focused for class prediction.

**Figure 11:**
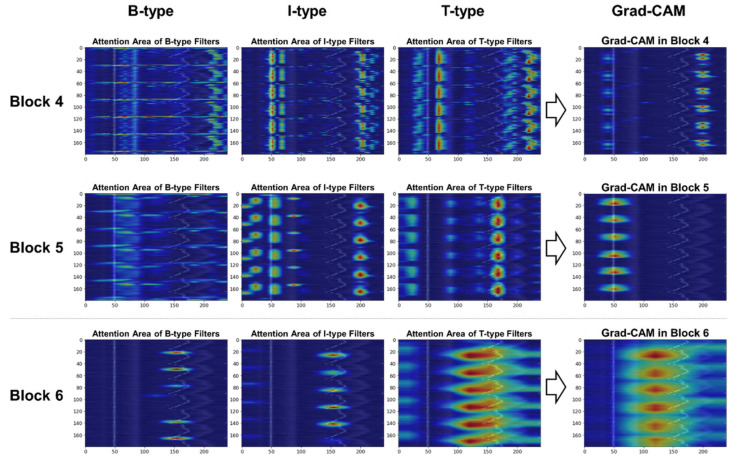
Comparison of filter-specific attention regions in blocks 4 to 6 for the normal class.

Figure 11 depicts a visualization example of the highlighted regions for the normal class according to each filter type in blocks 4 to 6. The importance of the specific regions in predicting a target class is expressed in colors ranging from red (high value) to blue (low value). The area of interest for the B-type filters moving across each row is revealed as a horizontal spread of highlights. For the I-type filters that perform convolution column-wise, the highlights are likely to appear in a vertical direction. Meanwhile, the heatmaps confirm that the T-type filters looked at areas wider than B- or I-type filters because they take into account the bi-direction (i.e., both rows and columns) for convolution. Based on the discriminative regions, the model classified the CS-ECM images as normal by looking at the R-peaks and their surroundings in blocks 4 and 5, and the patterns between the two adjacent R-peaks in block 6. In particular, in block 6, the T-type filters appeared to be more important than other filter types in normal classification.

**Figure 12:**
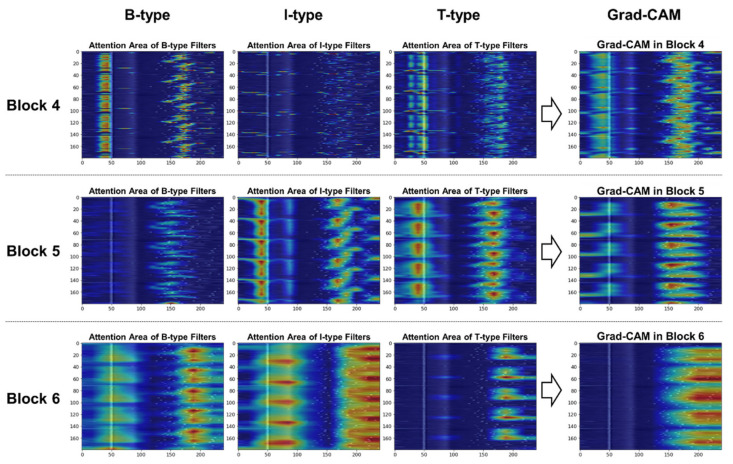
Comparison of filter-specific attention regions in blocks 4 to 6 for the AF class.

Figure 12 depicts a visual comparison of the important regions in blocks 4 to 6 when classifying the AF class. In Block 4, the left region of R-peaks receives the most attention from B-type and T-type filters; that is, the model was looking at the absence of P waves. In blocks 5 and 6, all three types of filters pay attention to the scattered patterns generated by the irregularly occurring R-peaks.

**Figure 13:**
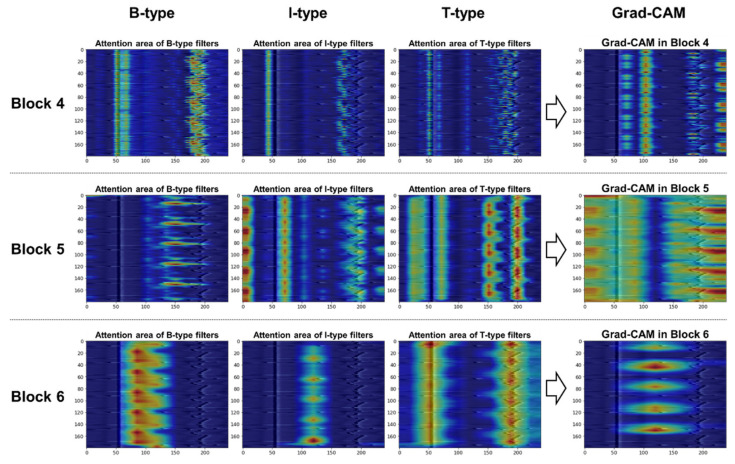
Comparison of filter-specific attention regions in blocks 4 to 6 for the other class.

Figure 13 illustrates how the contribution of each filter type in the other class differs from that of normal and AF classes. From the Grad-CAM heatmaps in block 6, unlike the previously mentioned case, in which the role of T-type filters in normal class prediction was greater than that of other types of filters, the B- and I-type filters appeared more important in other class prediction. The highlights on the location of random R-peaks are shown in the middle of the CS-ECM image, which is different from the normal or AF classes.

By observing the visualization of Grad-CAM, we can verify class-discriminative regions in the CS-ECM image, which are used to classify a certain class. A comprehensive comparison of qualitative interpretations showed that our proposed BIT-CNN model automatically identifies and localizes the most relevant regions of each heartbeat or inter-beat patterns corresponding to a certain class in the CS-ECM image.

## 4. Conclusions

This study presented a novel deep learning approach to classify multi-class rhythms from short single-lead ECG recordings, which is distinct from the state-of-the-art deep learning-based ECG classification studies. For this purpose, we reconstructed variable-length ECG recordings to fixed-size CS-ECM images, and fed them to the BIT-CNN for automatic feature learning and classification.

Our model effectively extracted features from the time-morphology representation with multiple convolution filters of different shapes and selectively paid attention to the salient parts using two types of attention mechanism. In addition, the 1 × 1 convolution strategy efficiently summarized the feature map channels to pass them to the next layer while reducing the overall network parameters. We also showed that the time-morphology representation is helpful for intuitively understanding and discovering interpretable features by simultaneously considering the shape and the rhythm of beats appearing in an arbitrarily short period.

Compared with some state-of-the-art methods for ECG classification using the PhysioNet/CinC Challenge 2017 dataset, we achieved a competitive performance with a relatively high interpretability and low computational cost. Our method is advantageous when applied in mobile health applications because it is lightweight and aids understanding by various types of users. In addition, the detection performance of the “other” rhythm (other types of arrhythmias) was slightly disappointing. Because we trained a limited number of parameters, we assume that our model had not learned sufficient information about the other class, which included many other types of abnormal rhythms compared to AF. Therefore, we will carry out further research to improve the performance of our approach, particularly for detection of non-AF. We also plan to apply our approach to the analyses of other physiological signals collected from other wearable measurements, and publicly available multi-lead ECG recordings, to expand the approach’s availability in the future.

## Figures and Tables

**Table 1:** Class distribution and statistics of the dataset.

Type	No. of Recordings	In %	Time Length (s)				
			Mean	SD	Max	Median	Min
Normal (N)	5076	59.52	32.11	9.97	60.95	30	9.05
AF (A)	758	8.89	32.34	12.32	60.21	30	9.99
Other (O)	2415	28.32	34.3	11.76	60.86	30	9.13
Noisy (P)	279	3.27	24.38	10.41	60	30	9.36
Total	8528	100	32.5	10.89	60.95	30	9.05

**Table 2:** Confusion matrix and performance scores (in %) of the BIT-CNN evaluated by a five-fold cross-validation.

Actual	Predicted				Sens. (Recall)	PPV (Precision)	F1-Score
	Normal	AF	Other	Noisy			
Normal	4611	23	354	88	90.84	88.66	89.73
AF	29	610	97	22	80.47	81.66	81.06
Other	524	103	1712	76	70.89	78.39	74.45
Noisy	37	11	21	210	75.27	53.03	62.22

**Table 3:** Classification performance of the proposed approach compared with the relevant CNN-based methods.

Methods	Input Length	Network	Validation	F_1N_	F_1A_	F_1O_	F_1P_	F_1_NAO_	F__NAOP_	Visual Interpretation
[56]	30 s	ResNet (34 layers)	5-fold CV	90.2	65.7	69.8	64.0	75.2	72.4	None
[57]	N/A	2D CNN with LSTM layer	5-fold CV	88.8	76.4	72.6	64.5	79.2	75.58	None
[54]	9, 15 s	DenseNet	5-fold CV	91	80	76	N/A	82	N/A	None
[55]	9–61 s	16-layer 1D residual CRNN	5-fold CV	91.9	85.8	81.6	N/A	86.4	N/A	None
[52]	30 s	1D CNN	5-fold CV	N/A	N/A	N/A	N/A	82.2	78.2	None
[53]	60.5 s	Modified ResNet	8:1:1 split	N/A	N/A	N/A	N/A	79.59	N/A	Included
[58]	9–61 s	Dense18+ for spectrogram	10-fold CV	89.29	79.18	72.25	52.50	80.24	73.31	Included
Proposed	9–61 s	Proposed BIT-CNN	5-fold CV	89.73	81.06	74.45	62.22	81.75	76.87	Included

## Data Availability

Not applicable.
